# Assessing alternative methods for unsupervised segmentation of urban vegetation in very high-resolution multispectral aerial imagery

**DOI:** 10.1371/journal.pone.0230856

**Published:** 2020-05-07

**Authors:** Allison Lassiter, Mayank Darbari

**Affiliations:** 1 Department of City and Regional Planning, University of Pennsylvania, Philadelphia, Pennsylvania, United States of America; 2 Department of Computer and Information Science, University of Pennsylvania, Philadelphia, Pennsylvania, United States of America; The University of the South Pacific, FIJI

## Abstract

To analyze types and patterns of greening trends across a city, this study seeks to identify a method of creating very high-resolution urban vegetation maps that scales over space and time. Vegetation poses unique challenges for image segmentation because it is patchy, has ragged boundaries, and high in-class heterogeneity. Existing and emerging public datasets with the spatial resolution necessary to identify granular urban vegetation lack a depth of affordable and accessible labeled training data, making unsupervised segmentation desirable. This study evaluates three unsupervised methods of segmenting urban vegetation: clustering with k-means using k-means++ seeding; clustering with a Gaussian Mixture Model (GMM); and an unsupervised, backpropagating convolutional neural network (CNN) with simple iterative linear clustering superpixels. When benchmarked against internal validity metrics and hand-coded data, k-means is more accurate than GMM and CNN in segmenting urban vegetation. K-means is not able to differentiate between water and shadows, however, and when this segment is important GMM is best for probabilistically identifying secondary land cover class membership. Though we find the unsupervised CNN shows high degrees of accuracy on built urban landscape features, its accuracy when segmenting vegetation does not justify its complexity. Despite limitations, for segmenting urban vegetation, k-means has the highest performance, is the simplest, and is more efficient than alternatives.

## Introduction

In many cities, urban vegetation is changing with the adoption of green infrastructure programs. In some cases, these are multibillion-dollar programs [1] that subsidize or create incentives for installing green infrastructure (e.g., parks, bioswales, street trees, and rain gardens) or replacing impervious surfaces (e.g., asphalt) with pervious ones (e.g., grasses). Despite scale and cost, however, there is often little empirical evaluation of programs due to challenges in data quality and attribution. With very high-resolution maps of vegetation structure through a city it would be possible to ask questions like: how are greening programs changing the quantity and type of green within the city? how does an individual parcel-owner’s participation in a greening program relate to neighborhood landscape change? how are greening programs driving ecological co-benefits, like habitat creation? To track patterns of urban greening across a city, including differential dynamics on public and private lands, this study examines if it is possible to leverage very high-resolution remotely sensed aerial imagery.

Most existing studies evaluate urban greenness at spatial resolutions coarser than the coverage of urban green infrastructure. Many use Landsat imagery at a spatial resolution of 30 meters or MODIS imagery at resolutions of 250–1000 meters [2–6]. Zhou et al. [7] find that Landsat underestimates vegetative cover relative to 2.5-meter SPOT/ALOS by approximately 10–25%, however, which they attribute to small changes in land cover. These small changes are typical of urban greening programs. Myint et al. [8] describe, to classify objects like landscape features, the spatial resolution of the imagery “needs to be at least one-half the diameter of the smallest object.”

Identifying small changes in vegetation calls for spatially high-resolution imagery, but using commercial imagery can be expensive to scale across a broad spatial extent or over time. Commercial imagery providers also often lack a historical archive, limiting possibilities for time-series analysis. Among publicly available, time-series data products in the United States, the broadband multispectral aerial imagery from the National Agricultural Imagery Program (NAIP) has the highest spatial resolution (0.6 or 1.0 meters). NAIP is the only publicly available data product with a spatial resolution sufficiently granular to delineate many urban vegetation features.

Thus far, there have been few studies that exclusively use the NAIP data product to identify urban vegetation without supplementary data. One notable exception is Basu et al. [9], who use NAIP spectral signatures with Conditional Random Fields, an unsupervised, machine learning technique, to identify tree canopy. They achieve a 74% accuracy across the state of California, spanning both urban and exurban spaces.

Within the urban environment, object-based landscape classification has been shown to resolve vegetation more accurately than pixel-based classifiers. After implementing an object-based classifier on 2.4-meter Quickbird imagery, Myint et al. [8] see classification accuracy improve over 20% (to 90.4%) compared to a pixel-based classifier. Mathieu, Freeman, and Aryal [10] find that using an object-based classifier and 2.5-meter IKONOS imagery results in a classification accuracy of 90.7%. Combining multiple imagery sources and fuzzy object-based classifiers, Li et al. [11] classify urban vegetation with 97.37% accuracy. Using NAIP imagery, Li et al. [12] create a 1-meter resolution land cover map with an object-based classifier supplemented with GIS parcel data (including land use attributes) for an overall accuracy of 91.86%. O’Neil-Dunne et al. [13] combine NAIP with LiDAR and thematic GIS data to identify urban tree canopy with up to 98% accuracy. Maxwell et al. [14] combine NAIP, derived spectral data, and thematic GIS data to map land cover types at 96.7% accuracy.

Using a supervised classifier and NAIP imagery, Robinson et al. [15] made the first high-resolution, nationwide land cover map. They combine 1-meter labeled NAIP data from the Chesapeake Bay, Maryland, 30-meter Landsat imagery and 30-meter National Landcover Dataset labels. Across all land covers in two test locations in Maryland and Iowa, they achieve a mean accuracy upwards of 90%.

While object-based and supervised learning methods are the gold standard for image classification, the challenge is that they require abundant training data. Sometimes these are hand-engineered features, but most useful is labeled imagery that has the same spectral and spatial resolution as the studied imagery. Acquiring good quality, labeled training data can be prohibitively expensive and time consuming. Robinson et al [15] note that the labeled NAIP data used as the basis of their study took 10 months to generate and cost $1.3 million. The burden of requiring training data means that supervised learning methods are difficult to implement on less commonly used or emerging datasets. With the ongoing proliferation of new cameras, sensors, and datasets, there will always be a shortcoming of labeled data. As a result, unsupervised learning methods will continue to be a critical complement to supervised methods, even as libraries of training data grow.

We evaluate unsupervised methods for segmenting sub-parcel urban vegetation. We start with spectral clustering methods: k-means with k-means++ seeding and a Gaussian Mixture Model (GMM). Then, we use a backpropagating convolutional neural network (CNN) with simple iterative linear clustering (SLIC) superpixels. While CNNs are frequently used in supervised learning problems [16], they are less common in unsupervised learning despite great potential [17, 18]. We compare k-means, GMM, and CNN segmentation by benchmarking classified data against internal validity metrics and hand-coded data.

## Materials and methods

### Study area and data

**Fig 1:**
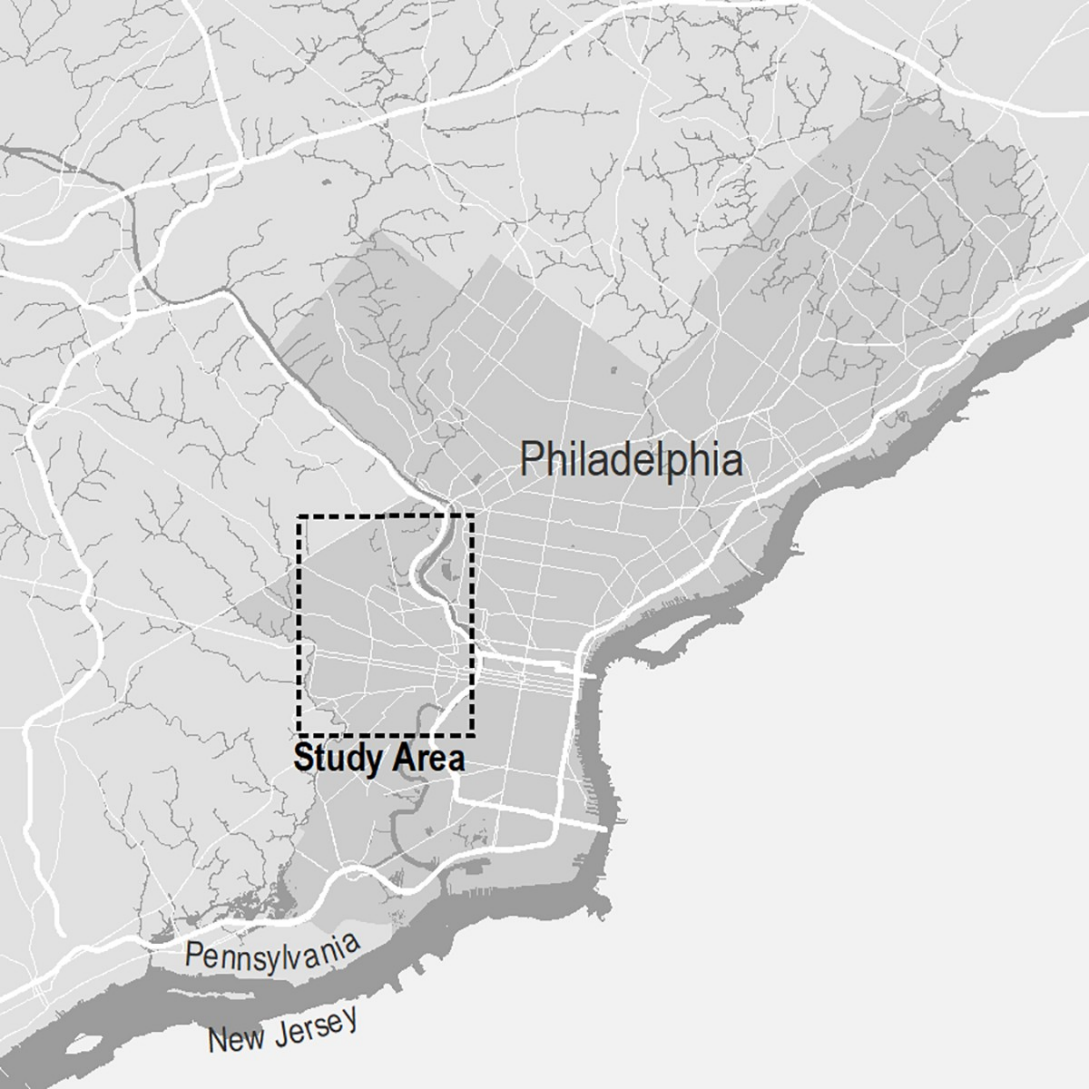
The study area in Philadelphia, Pennsylvania, USA. Map made by the authors with data from opendataphilly.org.

The NAIP imagery evaluated in this study represents a region of approximately 40 square kilometers in west Philadelphia, Pennsylvania, USA (Fig 1). The area includes urban land uses (commercial, industrial, and residential) and undeveloped regions (parks, grassland, unmanaged soil, and open water), providing a diversity of land uses and land cover classes.

The image was acquired on August 18, 2015. It has a spatial resolution of 1 meter and four channels: blue (435–495 nanometers), green (525–585 nanometers), red (435–495 nanometers), and near infrared (808–882 nanometers). The radiometric resolution of the datasets is eight bits and the data are radiometrically calibrated, but not atmospherically corrected. Maxwell et al [19] review NAIP imagery in the context of land cover classification, noting that one of the most significant challenges when using NAIP is inconsistent digital numbers across images. We focus our study on one image, which we subdivide into 800x800 pixel tiles to improve processing times.

We examine the data through its four native channels and six indices shown in the literature to effectively identify vegetation (Table 1). The Normalized Difference Vegetation Index (NDVI) and Enhanced Vegetation Index (EVI) enhance the vegetation signal. The Soil Adjusted Vegetation Index (SAVI) and Crust Index (CI) minimize background soil effects. The Atmospherically Resistant Vegetation Index (ARVI) and Visual Atmospheric Resistance Index (VARI) reduce atmospheric and topographic effects. With the indices, the final dimensions of the image are 7200 rows x 5600 columns x 10 channels.

**Table 1:** Vegetation indices.

Index	Formula
Normalized Difference Vegetation Index [20]	$NDVI=\frac{NIR-Red}{NIR+Red}$
Enhanced Vegetation Index [21]	$EVI=\frac{2.5*(NIR-Red)}{\left(NIR+6*Red+7.5*Blue+1\right)}$
Soil Adjusted Vegetation Index [22]	$SAVI=\frac{\left(NIR-Red)*(1+L\right)}{\left(NIR+Red+L\right)}$
	where *L* is a constant
Crust Index [23]	$CI=1-\frac{\left(Red-Blue\right)}{\left(Red+Blue\right)}$
Atmospherically Resistant Vegetation Index [24]	$ARVI=\frac{\left(NIR-(2*Red-Blue)\right)}{\left(NIR+(2*Red+Blue)\right)}$
Visual Atmospheric Resistance Index [25]	$VARI=\frac{\left(Green-Red\right)}{\left(Green+Red+Blue\right)}$

## Methods

The analysis proceeds in three steps. First, we mask out impervious surfaces (e.g., roads and buildings). Then, focusing on the pervious regions, we further segment the image. We apply k-means, GMM, and a CNN to the NAIP images, creating five major segments. We assign a land cover class to each segment: (1) water/shadow, (2) soil/ground, (3) shrub/tree, (4) grass, and (5) other. Finally, we evaluate the segmentation patterns for accuracy.

### Masking

To reduce the risk of spurious associations in the vegetation analysis, we mask out impervious surfaces. We use k-means clustering [26] with k-means++ seeding [27] to create two clusters based on NDVI values. One of the drawbacks of k-means is it requires an initial random seeding to place the first iteration of centroids from which to calculate the within-group and between-group variances. It is possible that the centroids may settle at local minima instead of the global minimum, resulting in poorly defined clusters. For more robust centroid seeding, rather than placing all centroids simultaneously, the k-means++ algorithm selects one centroid at random and then allocates subsequent centroids based on the prior centroids, given the first one. It is still subject to error, but less so than traditional k-means seeding. To model differences between traditional seeding and k-means++, we use an inertia score, which is the sum of squared distances to the closest cluster. We find that k-means++ leads to a lower inertia score, indicating a better fit (results available upon request).

### Segmentation

#### Clustering with k-means

We use k-means with k-means++ seeding to define five hard clusters based on the original four bands of the image and six vegetation indices. K-means scales efficiently to higher dimensionality and additional channels present little computational burden.

We implement k-means in python using the PySpark library, a big data processing framework. We define three parameters: five clusters, a Euclidean distance metric, and a maximum of twenty iterations.

#### Clustering with GMM

We use GMM to define five soft clusters based on the ten channels. Soft clustering calculates the probability that each pixel falls within all clusters [28]. GMM conveys some advantages over k-means. Firstly, its cluster definitions are less vulnerable to outliers. Like k-means, centroid placement with GMM is iterative. Unlike k-means, GMM uses all pixel information to update centroid locations, not just the information within a given cluster. Secondly, because GMM assigns each pixel to all classes with a probability of membership, the secondary class membership may be useful in some image interpretation problems. In particular, examining the second-class membership after water/shadow may be a useful method for “seeing under” or removing shadows in some environments [29]. The drawback, however, is that GMM is more computationally intensive and does not scale well to high dimensionality.

We implement GMM in python using the PySpark library. We define five clusters, use posterior probability as the convergence metric, and set the maximum number of iterations to twenty.

#### Using a CNN to delineate vegetation types

Instead of defining class membership through clustering, the CNN assigns spatially continuous pixels and pixels associated with similar features to the same class, while minimizing the number of unique classes. We adapt the algorithm proposed by Kanezaki [18] for a backpropagating CNN, which is specifically designed for unsupervised segmentation.

**Fig 2:**
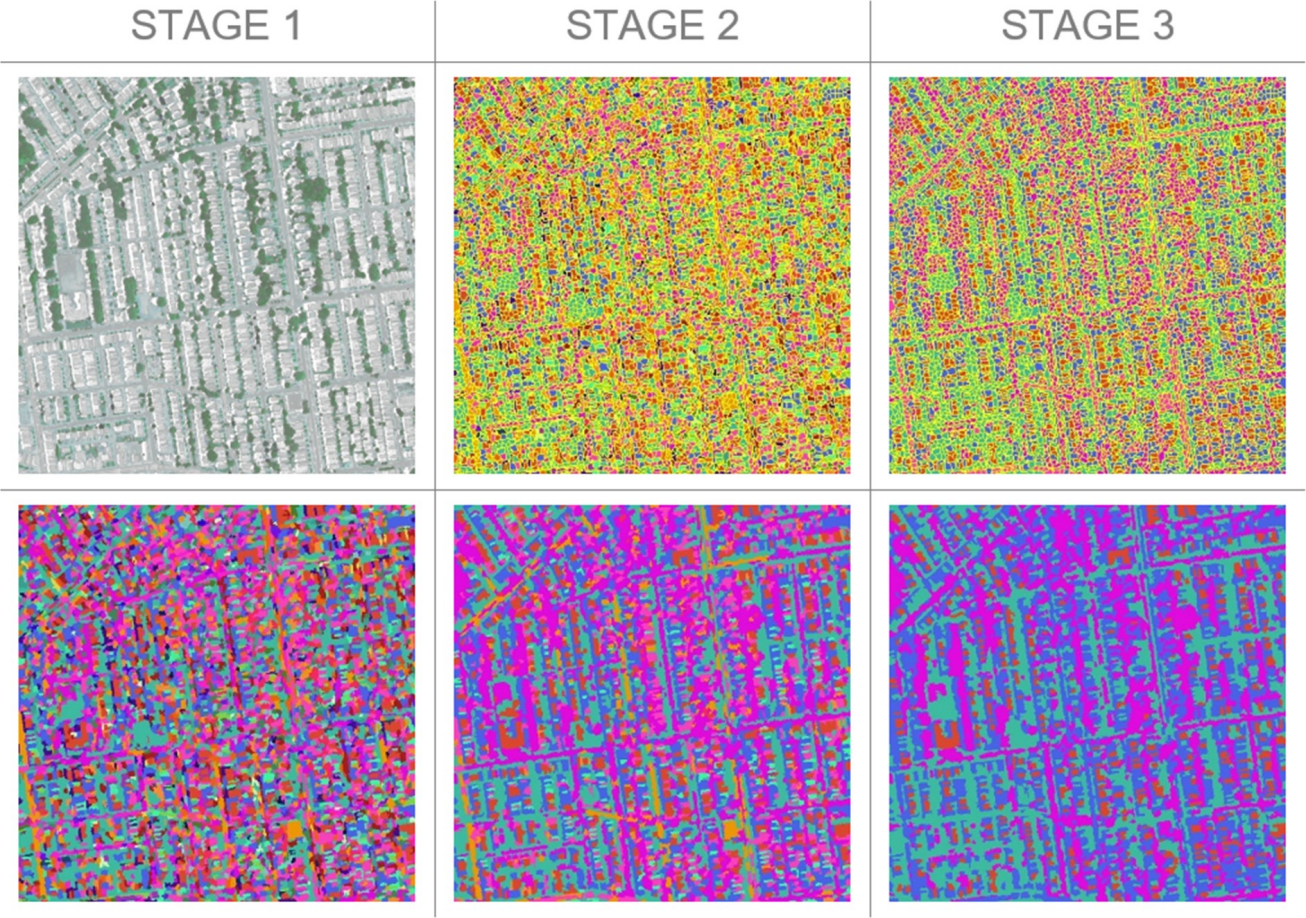
Progression of the CNN segmentation algorithm. Stage 1 transforms the original image (top) to a feature map (bottom). Stage 2 develops the segmentation map after applying the SLIC superpixels. Stage 3 refines the segmentation map after errors are backpropagated through the CNN. NAIP Digital Ortho Photo Image courtesy of USDA-FSA-APFO Aerial Photography Field Office.

Fig 2 describes the segmentation of the image as it moves through the CNN. In Stage 1, the CNN applies rectified linear unit and batch normalization operations to each pixel in the input image (Stage 1, top), transforming it to a feature map (Stage 1, bottom). The CNN does this by first assigning a multi-dimensional vector to each pixel, then using an argmax classifier to assign a cluster label to each pixel based on the largest feature index. In Stage 2, the CNN applies SLIC superpixels to the feature map developed in the first stage, assigning each pixel the same label as the majority within its superpixel. This produces a new segmentation map (Stage 2, bottom). In Stage 3, the CNN calculates errors, which are propagated back through the network. The CNN repeats Stages 1–3 iteratively, reducing the number of cluster groups (initially, approximately 50) to five (Stage 3, bottom).

We run the CNN in python using the PyTorch library and the skimage library’s implementation of SLIC. We parameterize the network architecture for the CNN to comprise three convolutional layers with each layer consisting of 100 convolutional filters. We set the learning rate to 0.1, Stochastic Gradient Descent as the optimizer, and the momentum parameter to 0.9. We use cross-entropy loss as the optimization function for the CNN. For the SLIC algorithm, we set the number of superpixels to 10,000 and the compactness of each superpixel to 10.

### Evaluation

#### Segment uniformity and disparity

Unsupervised learning is most useful when there is inadequate training data, but this lack of labeled data also makes it challenging to evaluate algorithmic performance. To overcome the problem of labeled data, Zhang et al. [30] use internal image-segment validity metrics to evaluate segmentation quality. They suggest four criteria:

1. Regions should be uniform and homogeneous with respect to some characteristic(s);
2. Adjacent regions should have significant differences with respect to the characteristic on which they are uniform;
3. Region interiors should be simple and without holes;
4. Boundaries should be simple, not ragged, and be spatially accurate.

The first two criteria, “characteristic” criteria, relate to the structure of segmented objects, while the last two, “semantic” criteria, relate to object legibility. Unlike characteristic criteria, semantic criteria are application- or object-dependent. Neither of the semantic criteria is applicable to vegetated land cover, so we only consider characteristic criteria.

The Davies-Bouldin Index [31] evaluates both characteristic criteria: intra-cluster uniformity and inter-cluster disparity. It is an internal evaluation scheme, where the validation of how well the clustering has been done is based on quantities and features inherent to the dataset. We apply the DBI to the k-means, GMM, and CNN image segmentations.

#### Benchmarking against classified data

To compare the output from the unsupervised image segmentation methods against labeled data, we hand-code a set of pixels. We label three tiles, or 1,920,000 pixels, as belonging to one of the five land cover classes (water/shadow, soil/ground, shrub/tree, grass, and other). Then, we generate evaluation metrics and confusion matrices to gauge the performance of each segmentation method for each landscape type.

## Results

### Masking

**Fig 3:**
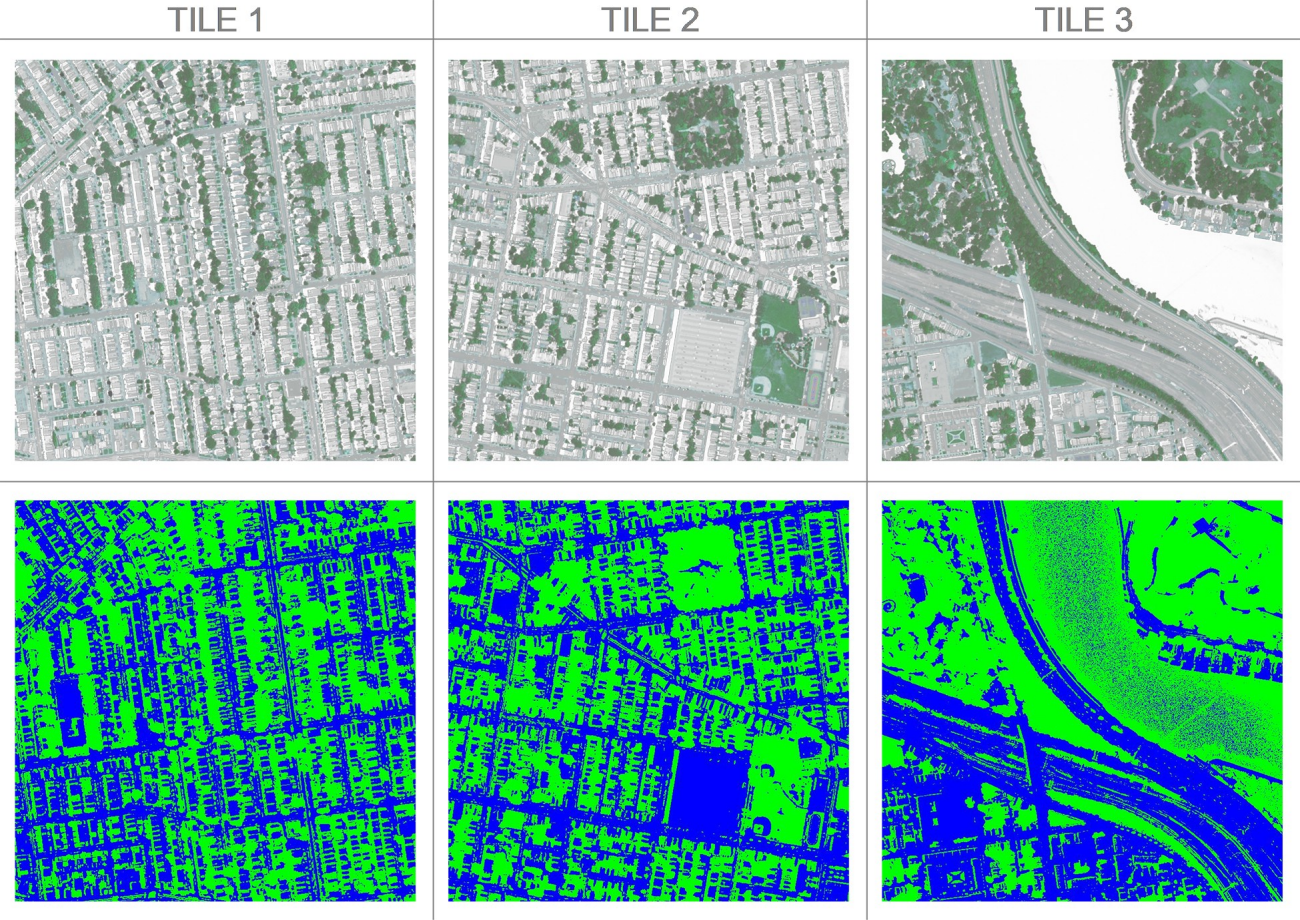
**Original image (top) and the vegetation mask (below).** Blue indicates the impervious region, green indicates the pervious (water/shadows, ground/soil, grass, trees/shrubs, and other) region. NAIP Digital Ortho Photo Image courtesy of USDA-FSA-APFO Aerial Photography Field Office.

The initial masking step removes all impervious surfaces and highly reflective pure water (not mixed with soil or vegetation) from the sample (Fig 3). All vegetation, ground/soil, and most water/shadow remain.

### Segmentation

**Fig 4:**
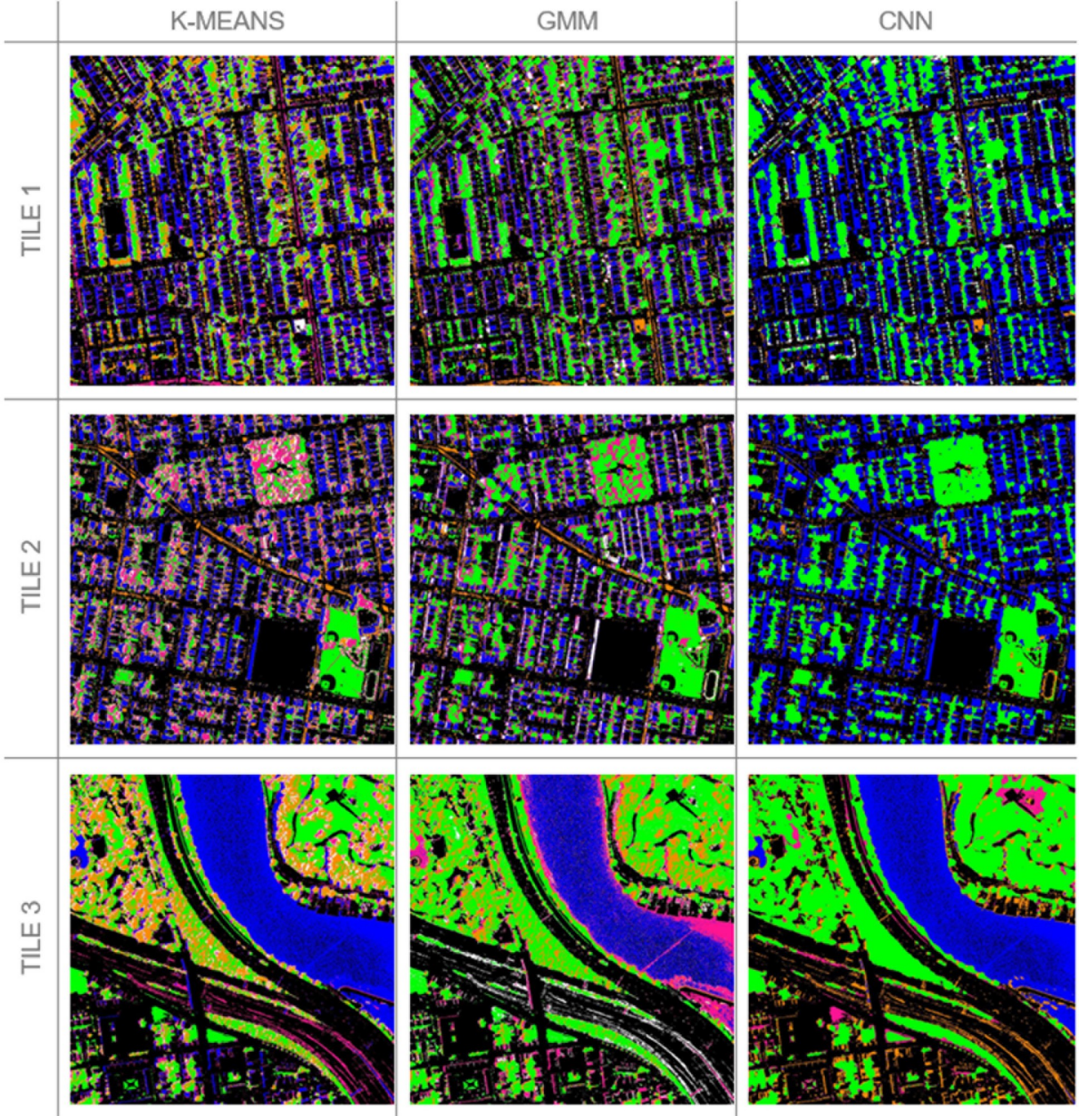
Selected tiles. Results of clustering: water/shadows (blue), ground/soil (pink), grass (green), trees/shrubs (orange), and other (white). Black is impervious regions masked from the vegetation analysis.

Fig 4 presents segmentation results. K-means and GMM exhibit different segmentation patterns, with GMM emphasizing grass and identifying some river water as ground/soil. CNN produces a segmentation pattern very different from the other two with far fewer small segments. It does not successfully represent objects like trees in the middle of grass or slivers of ground/soil.

### Evaluation

#### Segment uniformity and disparity

Table 2 presents Davies-Bouldin Index results for each segmentation method. A lower index number indicates less in-class variance and greater between-class variance. For k-means, the index values range from 0.63 to 0.71. For GMM, index values range from 1.53 to 5.24. For CNN, index values range 1.18 to 2.85. According to the DBI, all tiles perform better with k-means than GMM or CNN. While there are some qualities to the image tiles that may lead to different DBI values, not all the differences in the imagery shown in Fig 4 are visually perceptible (the NIR band is not displayed).

**Table 2:** Davies-Bouldin Index results for 800x800 NAIP image tiles. A lower index number indicates lower in-class variance and greater between-class variance (overall better performance).

Tile	DBI for k-means	DBI for GMM	DBI for CNN
1	0.71	1.82	2.85
2	0.70	5.24	2.14
3	0.63	1.53	1.18
Mean	0.68	2.86	2.06

#### Benchmarking against classified data

Compared to the hand-coded data, k-means image segmentation outperforms both GMM and CNN, though there is variability from tile to tile (Table 3). K-means has the strongest performance on all the tiles, with an average accuracy of 80%. It performs best on Tile 3 (81%) and worst on Tile 1 (79%). Conversely, GMM performs best on Tile 1 (55%) and worst on Tile 3 (45%). Like k-means, CNN performs its best (69%) on Tile 3 but is only 28% accurate on Tile 2.

**Table 3:** Accuracy of the unsupervised learning methods when compared against hand-coded data over all pervious land cover classes.

Tile	k-means	GMM	CNN
1	0.79	0.55	0.51
2	0.80	0.47	0.28
3	0.81	0.45	0.69
Mean	0.80	0.48	0.49

Not only is there variation in accuracy by tile, there is also variation by land cover class. Evaluation metrics by land cover class (accuracy, recall, precision and F1 scores) are available in Table 4. K-means classifies vegetation the best among the three methods, with grass at 80% accuracy and trees/shrubs at 78%. GMM is the weakest performing method, with grass at 26% accuracy and trees/shrubs at 55%. CNN segments grass at 74% accuracy and trees/shrubs with 49%. Confusion matrices, available in Table 5, reveal which classes the segmentation algorithms regularly mistake. For example, k-means regularly identifies water/shadow as grass, but GMM never does.

**Table 4:** Evaluation metrics. Accuracy is the true positives (correct predictions) over the total number of predictions. Recall (user’s accuracy) is the fraction of true positives over the sum of true positives and false negatives. Precision (producer’s accuracy) is the fraction of true positives over the sum of true positives and false positives. The F1-score ranges from 0–1, where 1 is the optimal balance of recall and precision.

k-means	Accuracy	Recall	Precision	F1-score
Ground/Soil	0.73	0.73	0.73	0.73
Water/Shadow	0.84	0.84	0.84	0.84
Grass	0.80	0.80	0.81	0.80
Trees/Shrubs	0.78	0.78	0.78	0.78
Other	0.78	0.78	0.78	0.78
GMM				
Ground/Soil	0.29	0.29	0.18	0.22
Water/Shadow	0.66	0.66	0.85	0.75
Grass	0.26	0.26	0.30	0.28
Trees/Shrubs	0.55	0.55	0.35	0.43
Other	0.18	0.18	0.32	0.23
CNN				
Ground/Soil	0.13	0.10	0.13	0.11
Water/Shadow	0.69	0.46	0.69	0.55
Grass	0.74	0.44	0.74	0.55
Trees/Shrubs	0.49	0.88	0.50	0.64
Other	0.33	0.46	0.33	0.39

**Table 5:** Confusion matrices comparing results from the unsupervised classification algorithms to the hand-coded training data (analyzing 1,920,000 pixels, with pixels eliminated by masking). The column contains the class to which each pixel belongs and the row is the predicted value.

k-means	Ground/Soil	Water/Shadow	Grass	Trees/Shrubs	Other
Ground/Soil	73497	6467	4214	9152	7082
Water/Shadow	6467	279844	13834	13072	16968
Grass	4855	13843	173213	14877	6814
Trees/Shrubs	8481	13263	16214	159341	6698
Other	7215	17258	7030	6603	137799
GMM					
Ground/Soil	29978	48461	4315	5879	76880
Water/Shadow	42	221292	0	1	36662
Grass	28477	3418	56075	78574	14456
Trees/Shrubs	20728	20960	148971	112072	14650
Other	21290	36544	5144	6519	32713
CNN					
Ground/Soil	9749	36573	63	1232	28254
Water/Shadow	17001	151324	815	3318	47644
Grass	9829	234	93762	17723	4846
Trees/Shrubs	49828	298	115913	178877	14075
Other	13508	142414	3934	2072	80815

## Discussion

The unsupervised segmentation methods evaluated in this study do not provide the same degree of accuracy for segmenting urban vegetation as has been achieved with some object-based and supervised methods [8, 10]. Even so, unsupervised k-means, GMM, and CNN methods are reasonable approaches for classifying some landscape features to create low-cost, scalable vegetation maps without training data. In particular, in this study area, k-means delineates urban vegetation with surprising accuracy compared to more complex alternatives (Table 4).

One of the challenges of the segmentation problem is that many pixels in the study area likely represent multiple land cover types. This is exemplified by the pixels in the ground/soil and water/shadow class, which could have been dropped in the masking step if the spectral signature of the land cover included in the pixels aligned more closely with impervious surfaces and pure water. Instead, k-means finds the spectral signatures of these pixels align better with vegetation. It is possible this is partly due to errors in evaluating accuracy; some of the hand-coded pixels may be misclassified as ground/soil since pixels representing dormant (non-photosynthetically active) vegetation, dead vegetation, and soil are often spectrally similar [12, 32]. However, the pixels in the middle of the river that are not dropped in the initial masking (Fig 3, Tile 3) illustrate the mixed pixel problem most clearly. We believe these pixels are not masked from the analysis because there are degrees of both sedimentation and eutrophication present in the river. Immediately downstream of the study area is an old, low-head dam (Fig 3, visible in the lower left-hand corner of Tile 3) and the right bank of the river is heavily populated with boaters. The water in this area likely contains solutes and material that make it more spectrally similar to ground/soil than reflective, pure water. In another study area, some of these mixed water pixels would be grouped into the initial impervious/pure water cluster that was dropped in the masking step; the initial masking is sensitive to the boundary conditions of the study area and mixed pixels align with a class based on the full distribution of spectral values in the study area.

**Fig 5:**
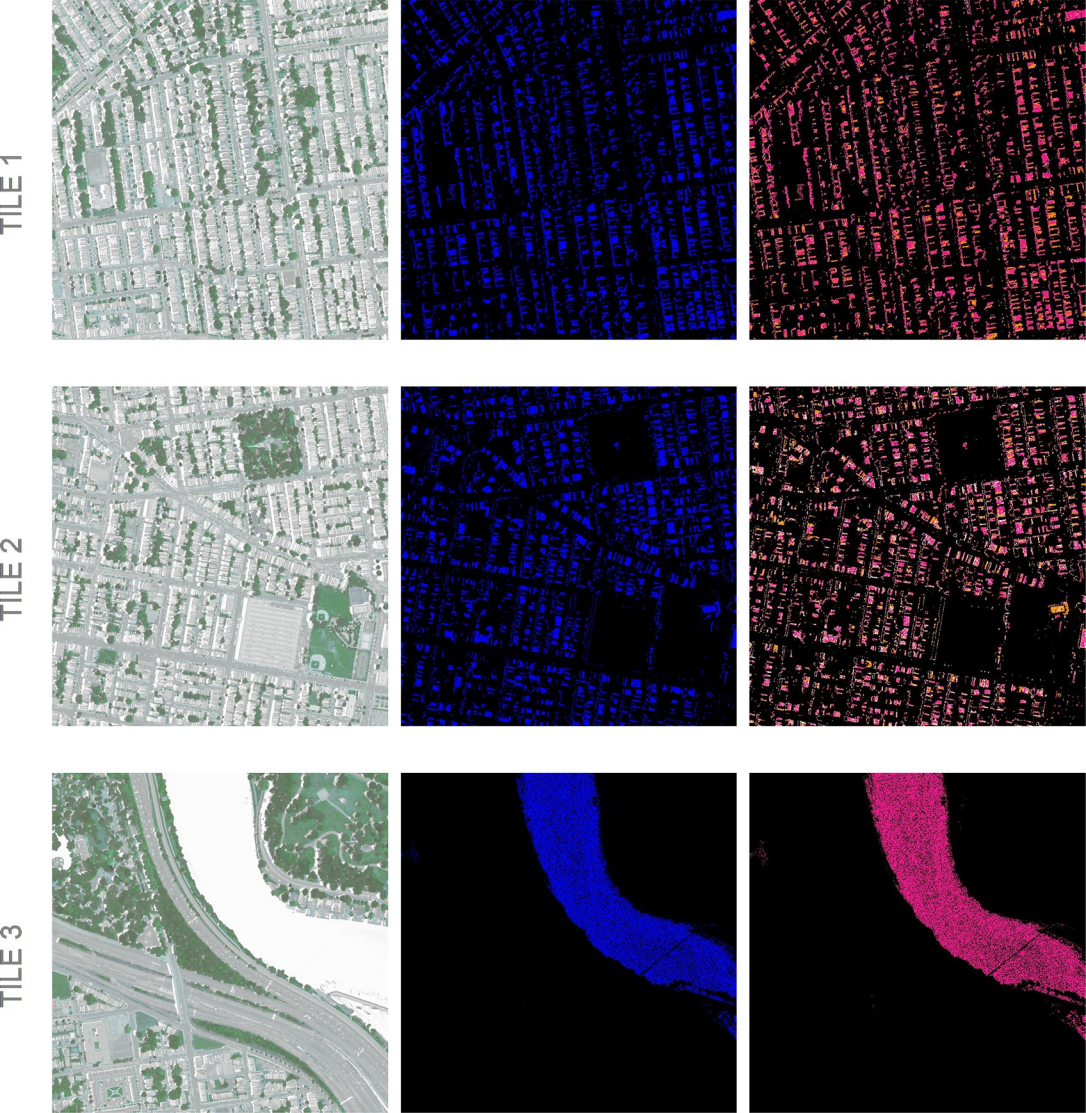
GMM segmentation of shadows. The first column shows the original imagery, the second column identifies water/shadows (blue), and the third column identifies the second most likely land cover class after water/shadows for each pixel: ground/soil (pink), trees/shrubs (orange), or other (white). Grass is never initially identified as water/shadow. NAIP Digital Ortho Photo Image courtesy of USDA-FSA-APFO Aerial Photography Field Office.

Water and shadows are grouped together as a class because they are difficult for k-means and CNN to distinguish. Spectrally, the dark color of the water with its underlying vegetation signals is very similar to shadowed vegetation. Though classifying water is not a primary focus of this study, separating the two would help identify the types of vegetation under shadow. To further evaluate the water/shadow segment, we visualize the second most probable class with GMM (Fig 5). Because GMM is a soft clustering method, even though a given pixel may most likely be within the water/shadows class, it will also be within all other land cover classes with some probability. The secondary land cover class gives insight into the land cover under shadow or mixed in the water. In Fig 5, GMM shows the secondary classes to be a combination of ground/soil and trees/shrubs. In the river in Tile 3, the secondary class is exclusively ground/soil.

To better understand the spectral channels driving the segmentation pattern in the clustering methods, we evaluate the relative contribution of each of the ten channels through a Gini index (Table 6). The Gini index gives a coefficient to each channel from zero to one. If all channels are equally represented in the final segments, the Gini coefficients would be zero. If a channel is more likely to be contained in a final segment, the coefficient approaches one. Among the channels evaluated in this study, NIR is the most important driver of the segmentation pattern, followed by green, red, and blue. All vegetation indices are much less important.

**Table 6:** Channel importance calculated using a Gini index [33].

Ranked importance	Channel	Gini Index
1	NIR	0.263
2	G	0.247
3	R	0.186
4	B	0.165
5	VARI	0.045
6	SAVI	0.025
7	NDVI	0.024
8	ARVI	0.020
9	CI	0.009
10	EVI	0.008

The Gini Index ranks the contribution of each channel to the final classification, given all channels present. Changing the set of available channels will change the Gini Index values. With the available channels, the Gini index indicates that segmenting through multi-dimensional clustering is an improvement over classifying through a single vegetation index, like NDVI. There remains opportunity to incorporate additional spectral channels and derived channels that are uncorrelated with RGB and NIR, like measures of texture, depth, or pixel neighborliness, to continue to improve classification accuracy of the clustering methods.

Using the same ten channels, GMM performs worse than k-means. While k-means has a mean classification accuracy of 80%, GMM has a mean accuracy of 48% (Table 3). The GMM results have lower DBI scores than k-means, too. Further, GMM is much more computationally intensive than k-means and does not scale efficiently. Its only advantage is its ability to detect secondary class membership.

The CNN, with a mean classification accuracy of 49%, slightly outperforms GMM, but performs poorly compared to k-means. This is unexpected, given the accuracy of many reported CNN implementations. To further evaluate CNN performance, we evaluate k-means, GMM, and the CNN against an existing labeled, very high-resolution urban land cover dataset [34] with three spectral bands (R, G, B) and find that the CNN (82.42% accuracy) performs significantly better than GMM (49.39% accuracy) and k-means (43.73% accuracy) when examining built urban features (Table 7).

**Table 7:** Accuracy results of k-means with k++ seeding, GMM, and the backpropagating CNN with SLIC superpixels applied to labeled RGB imagery.

Labeled urban feature	k-means	GMM	CNN
River	0.42	0.53	0.86
Mobilehomepark	0.24	0.22	0.61
Harbor	0.51	0.56	0.87
Golfcourse	0.46	0.65	0.87
Baseballdiamond	0.64	0.71	0.93
Overpass	0.46	0.61	0.87
Tenniscourt	0.22	0.22	0.75
Intersection	0.39	0.47	0.81
Airplane	0.67	0.64	0.93
Parkinglot	0.59	0.62	0.88
Sparseresidential	0.40	0.46	0.75
mediumresidential	0.34	0.39	0.72
Denseresidential	0.32	0.30	0.73
Beach	0.43	0.45	0.87
Freeway	0.50	0.59	0.92
Storagetanks	0.41	0.49	0.80
MEAN	0.44	0.49	0.82

The much higher accuracy on built urban features is consistent with the more common use of CNNs for delineating constructed urban features, like roads and buildings [35, 36], which have higher internal homogeneity and stronger edges. One of the features of many CNNs, including the one in this study, is the use of SLIC superpixels. The superpixels force internal homogeneity onto the segments. Internal homogeneity is usually a desirable attribute of a segment because it reduces salt-and-pepper effects, but this is less applicable to patchy vegetation. In contrast, k-means or GMM does not account for neighborliness when assigning clusters, allowing for salt-and-pepper effects. While the CNN shows far better accuracy on built urban features with three-band imagery, for urban vegetation on multispectral imagery, the backpropagating CNN with SLIC superpixels does not offer sufficiently better performance to justify its much more complex architecture.

One of the most significant challenges of this study is defining the best evaluation criteria with which to compare algorithmic performance. There is still further work to be done on developing evaluation criteria for unsupervised segmentation and classification. DBI and benchmarking against hand-coded data sometimes give contradictory results. For k-means, DBI indicates that its best performance is on Tile 3, while benchmarking indicates its worst performance is on Tile 3. For GMM, DBI results indicate that it performs very poorly on Tile 2, but benchmarking indicates its performance is on par with the other tiles. Because DBI measures segment uniformity and disparity, it may not be an adequate metric in the context of complex, heterogeneous urban vegetation, where segments may be neither uniform nor distinct.

Yet, despite shortcomings of DBI, an equally significant weakness is generating accurate labeled data for benchmarking. When hand-coding data, we can only visualize three bands simultaneously. A false-color composite can be used to visualize NIR, which the Gini Index indicates is very important in determining class membership (Table 6), but it is still difficult to visually separate all land cover types. While the advantage of hand-coding is that we implicitly incorporate shape into object labeling, perceiving objects like trees, some variation (e.g., between grass and soil) is more difficult to visually distinguish. Further, NAIP is still sufficiently coarse that it is difficult to distinguish objects less than 2 meters in diameter (following Myint et al [8]’s discussion of image resolution and object recognition). As a result, the labels we create for the data may not be perfectly accurate. This issue is echoed in a study from the California Data Collaborative [37], which compared commercially classified residential landscape data to hand-coded NAIP imagery, finding low rates of agreement among algorithms and hand-coding. But the California Data Collaborative also found low rates of agreement among different hand-coders reviewing the same parcels. The discrepancy between hand-coded data reveals that even though NAIP imagery offers the highest spatial resolution for publicly available data, it is coarse enough that it can be difficult for hand-coders to reliably classify parcel-level land cover. Physically ground-truthed data would provide a better benchmark but is difficult to scale.

To further evaluate algorithmic performance, segmentation and classification should be tested across a greater variety of biophysical and urban conditions. It is likely that each method will be useful in certain contexts, when segmenting particular urban landscape conditions. Additional channels may improve k-means and GMM performance. Alternative CNN architectures and parameters may improve CNN performance.

It is possible that the most efficacious unsupervised segmentation and classification method for identifying small and patchy urban vegetation will combine aspects of all three algorithms evaluated in this study. There is opportunity to consider how principals of GMM (e.g., “seeing” under shadows) and k-means (e.g., ragged boundary definition through many channels) could be brought into a CNN architecture. Evaluating algorithmic performance will remain an ongoing challenge, however, especially in shadowed regions. It is likely that data fusion will be necessary, up-sampling or down-sampling existing labeled data [15, 38]. If high-quality labeled data is available, however, it would be better to use supervised methods. It will always be most useful to apply unsupervised methods when there is an insufficient amount of labeled data.

## Conclusion

Though supervised learning methods are currently popular and can be very accurate for delineating some urban features, the burden of training data constrains the use of less common or emerging data sets with no available training data. We examine unsupervised segmentation of urban vegetation features from NAIP multispectral aerial imagery. We use two clustering methods, k-means and GMM, and a backpropagating CNN with SLIC superpixels. Each method has advantages: k-means with k-means++ seeding has the highest levels of segmentation accuracy for most landscape types and is both easy and efficient to implement; GMM provides the best information through shadowing; and the CNN, while underperforming on urban vegetation, has high accuracy on built features with hard edges and internal homogeneity. Yet, for segmenting urban vegetation from multispectral imagery without training data, k-means is the most accurate, simplest, and most efficient.

## References

[1] U.S. Environmental Protection Agency. Keeping Raw Sewage and Contaminated Stormwater Out of Our Nation's Waters, Status of Civil Judicial Consent Decrees Addressing Combined Sewer Systems: EPA National Enforcement Initiative; 2017. Available from: https://www.epa.gov/sites/production/files/2017-05/documents/epa-nei-css-consent-decree-tracking-table-050117.pdf.

[2] Gan M, Deng J, Zheng X, Hong Y, Wang K. Monitoring urban greenness dynamics using multiple endmember spectral mixture analysis. PloS One. 2014;9(11):e112202 10.1371/journal.pone.0112202 25375176 PMC4223042

[3] Dallimer M, Tang Z, Bibby PR, Brindley P, Gaston KJ, Davies ZG. Temporal changes in greenspace in a highly urbanized region. Biol Lett. 2011;7(5):763–6. 10.1098/rsbl.2011.0025 21429910 PMC3169039

[4] Johnson TD, Belitz K. A remote sensing approach for estimating the location and rate of urban irrigation in semi-arid climates. J Hydrol. 2012;414–415:86.

[5] Zhou X, Wang Y. Spatial–temporal dynamics of urban green space in response to rapid urbanization and greening policies. Landscape Urban Plan. 2011;100(3):268–77.

[6] Zhu Z, Fu Y, Woodcock CE, Olofsson P, Vogelmann JE, Holden C, et al Including land cover change in analysis of greenness trends using all available Landsat 5, 7, and 8 images: A case study from Guangzhou, China (2000–2014). Remote Sens Environ. 2016;185:243–57.

[7] Zhou W, Wang J, Qian Y, Pickett STA, Li W, Han L. The rapid but “invisible” changes in urban greenspace: A comparative study of nine Chinese cities. Sci Total Environ. 2018;627:1572–84. 10.1016/j.scitotenv.2018.01.335 30857118

[8] Myint SW, Gober P, Brazel A, Grossman-Clarke S, Weng Q. Per-pixel vs. object-based classification of urban land cover extraction using high spatial resolution imagery. Remote Sens Environ. 2011;115(5):1145–61.

[9] Basu S, Ganguly S, Nemani RR, Mukhopadhyay S, Zhang G, Milesi C, et al A semiautomated probabilistic framework for tree-cover delineation from 1-m NAIP imagery using a high-performance computing architecture. IEEE Trans Geosci Remote Sens. 2015;53(10):5690–708.

[10] Mathieu R, Freeman C, Aryal J. Mapping private gardens in urban areas using object-oriented techniques and very high-resolution satellite imagery. Landscape Urban Plan. 2007;81(3):179–92.

[11] Li W, Radke J, Liu D, Gong P. Measuring detailed urban vegetation with multisource high-resolution remote sensing imagery for environmental design and planning. Environment and Plann B. 2012;39(3):566–85.

[12] Li X, Myint SW, Zhang Y, Galletti C, Zhang X, Turner BL. Object-based land-cover classification for metropolitan Phoenix, Arizona, using aerial photography. Int J Appl Earth Obs. 2014;33:321–30.

[13] O'Neil-Dunne J, MacFaden S, Royar A. A Versatile, Production-Oriented Approach to High-Resolution Tree-Canopy Mapping in Urban and Suburban Landscapes Using GEOBIA and Data Fusion. Remote Sens-Basel. 2014;6(12).

[14] Maxwell AE, Strager MP, Warner TA, Ramezan CA, Morgan AN, Pauley CE. Large-Area, High Spatial Resolution Land Cover Mapping Using Random Forests, GEOBIA, and NAIP Orthophotography: Findings and Recommendations. Remote Sens-Basel. 2019;11(12):1409.

[15] Robinson C, Hou L, Malkin K, Soobitsky R, Czawlytko J, Dilkina B, et al. Large Scale High-Resolution Land Cover Mapping with Multi-Resolution Data. In Proceedings of the IEEE Conference on Computer Vision and Pattern Recognition 2019 Jun (pp. 12726–12735).

[16] Alom MZ, Taha TM, Yakopcic C, Westberg S, Sidike P, Nasrin MS, et al. The history began from AlexNet: a comprehensive survey on deep learning approaches. arXiv:1803.01164. 2018.

[17] Kanezaki A. Unsupervised image segmentation by backpropagation. In 2018 IEEE International Conference on Acoustics, Speech and Signal Processing. 2018 Apr 15 (pp. 1543–1547).

[18] Zhang C, Qiu F. Hyperspectral image classification using an unsupervised neuro-fuzzy system. J Appl Remote Sens. 2012;6(1):063515.

[19] Maxwell A, Warner T, Vanderbilt B,C., Ramezan C,A. Land Cover Classification and Feature Extraction from National Agriculture Imagery Program (NAIP) Orthoimagery: A Review. Photogramm Eng Rem S. 2017;83:737–747.

[20] Tucker CJ. Red and photographic infrared linear combinations for monitoring vegetation. Remote Sens Environ. 1979;8(2):127–50.

[21] Huete A, Didan K, Miura T, Rodriguez EP, Gao X, Ferreira LG. Overview of the radiometric and biophysical performance of the MODIS vegetation indices. Remote Sens Environ. 2002;83(1–2):195–213.

[22] Huete AR. A soil-adjusted vegetation index (SAVI). Remote Sens Environ. 1988;25(3):295–309.

[23] Karnieli A. Development and implementation of spectral crust index over dune sands. Int J Remote Sens. 1997;18(6):1207–20.

[24] Kaufman YJ, Tanre D. Atmospherically resistant vegetation index (ARVI) for EOS-MODIS. IEEE Trans Geosci Remote Sens. 1992;30(2):261–70.

[25] Gitelson AA, Kaufman YJ, Stark R, Rundquist D. Novel algorithms for remote estimation of vegetation fraction. Remote Sens Environ. 2002;80(1):76–87.

[26] Lloyd S. Least squares quantization in PCM. IEEE Trans Inf Theory. 1982;28(2):129–37.

[27] Arthur D, Vassilvitskii S. k-means: The advantages of careful seeding. Proceedings of the eighteenth annual ACM-SIAM symposium on Discrete algorithms; Society for Industrial and Applied Mathematics; 2007.

[28] Permuter H, Francos J, Jermyn IH. Gaussian mixture models of texture and colour for image database retrieval. In 2003 IEEE International Conference on Acoustics, Speech, and Signal Processing (ICASSP'03). 2003 Apr 6 (Vol. 3, pp. III-569).

[29] Martel-Brisson N, Zaccarin A. Moving cast shadow detection from a gaussian mixture shadow model. In 2005 IEEE Computer Society Conference on Computer Vision and Pattern Recognition (CVPR'05). 2005 (Vol. 2, pp. 643–648).

[30] Zhang H, Fritts JE, Goldman SA. Image segmentation evaluation: A survey of unsupervised methods. Comput Vis Image Und. 2008;110(2):260–80.

[31] Davies DL, Bouldin DW. A cluster separation measure. IEEE Trans Pattern Anal Mach Intell. 1979(2):224–7. 21868852

[32] Wetherley EB, Roberts DA, McFadden JP, Wetherley EB, Roberts DA, McFadden JP. Mapping spectrally similar urban materials at sub-pixel scales. Remote Sens Enviro. 2017;195:170–83.

[33] Raileanu LE, Stoffel K. Theoretical comparison between the gini index and information gain criteria. Ann Math Artif Intel. 2004;41(1):77–93.

[34] Yang Y, Newsam S. Bag-of-visual-words and spatial extensions for land-use classification. Proceedings of the 18th SIGSPATIAL international conference on advances in geographic information systems; ACM; 2010.

[35] Laddha A, Kocamaz MK, Navarro-Serment LE, Hebert M. Map-supervised road detection. In 2016 IEEE Intelligent Vehicles Symposium (IV). 2016 Jun 19 (pp. 118–123).

[36] Zhang Q, Wang Y, Liu Q, Liu X, Wang W. CNN based suburban building detection using monocular high resolution Google Earth images. In 2016 IEEE International Geoscience and Remote Sensing Symposium (IGARSS). 2016 Jul 10 (pp. 661–664).

[37] California Data Collaborative. Independent Landscape Area Classification Accuracy Assessment—Final Results. 2016.

[38] Khan A, Hansen M, Potapov P, Adusei B, Pickens A, Krylov A, et al Evaluating Landsat and Rapideye data for winter wheat mapping and area estimation in Punjab, Pakistan. Remote Sens-Basel 2018;10(4):489.

